# Ethanolamine Influences Human Commensal Escherichia coli Growth, Gene Expression, and Competition with Enterohemorrhagic E. coli O157:H7

**DOI:** 10.1128/mBio.01429-18

**Published:** 2018-10-02

**Authors:** Carol A. Rowley, Christopher J. Anderson, Melissa M. Kendall

**Affiliations:** a Department of Microbiology, Immunology, and Cancer Biology, University of Virginia School of Medicine, Charlottesville, Virginia, USA; University of Texas Health Science Center

**Keywords:** ethanolamine, metabolism, microbiota, signaling

## Abstract

A core principle of bacterial pathogenesis is that pathogens preferentially utilize metabolites that commensal bacteria do not in order to sidestep nutritional competition. The metabolite ethanolamine (EA) is well recognized to play a central role in host adaptation for diverse pathogens. EA promotes growth and influences virulence during host infection. Although genes encoding EA utilization have been identified in diverse bacteria (nonpathogenic and pathogenic), a prevailing idea is that commensal bacteria do not utilize EA to enhance growth, and thus, EA is a noncompetitive metabolite for pathogens. Here, we show that EA augments growth of two human commensal strains of Escherichia coli. Significantly, these commensal strains grow more rapidly than, and even outcompete, the pathogen enterohemorrhagic E. coli O157:H7 specifically when EA is provided as the sole nitrogen source. Moreover, EA-dependent signaling is similarly conserved in the human commensal E. coli strain HS and influences expression of adhesins. These findings suggest a more extensive role for EA utilization in bacterial physiology and host-microbiota-pathogen interactions than previously appreciated.

## Abstract (precis)

The microbiota protects the host from invading pathogens by limiting access to nutrients. In turn, bacterial pathogens selectively exploit metabolites not readily used by the microbiota to establish infection. Ethanolamine has been linked to pathogenesis of diverse pathogens by serving as a noncompetitive metabolite that enhances pathogen growth as well as a signal that modulates virulence. Although ethanolamine is abundant in the gastrointestinal tract, the prevailing idea is that commensal bacteria do not utilize EA, and thus, EA utilization has been particularly associated with pathogenesis. Here, we provide evidence that two human commensal Escherichia coli isolates readily utilize ethanolamine to enhance growth, modulate gene expression, and outgrow the pathogen enterohemorrhagic E. coli. These data indicate a more complex role for ethanolamine in host-microbiota-pathogen interactions.

## OBSERVATION

The microbiota plays essential roles in human health. For example, the microbiota functions as a barrier against invading pathogens by limiting access to nutrients (1). Significantly, bacterial pathogens have evolved to exploit specific host- and microbiota-derived metabolites to sidestep nutritional competition and control expression of virulence traits (1). For instance, ethanolamine (EA) is abundant in the gastrointestinal (GI) tract due to the turnover of bacterial and epithelial cells (EA is a breakdown product of the cell membrane lipid phosphatidylethanolamine) as well as through the diet (2). EA utilization plays a central role in host adaptation for a diverse range of pathogens, including opportunistic pathogens (3, 4). EA can serve as a carbon, nitrogen, and/or energy source to promote growth as well as a signal to influence virulence during host infection (5–11). Genes encoding EA utilization are carried in the ethanolamine utilization (*eut*) locus (12). In the *Enterobacteriaceae*, the *eut* locus encodes the transcription factor EutR. EutR senses EA and vitamin B_12_ to directly activate *eut* transcription (13, 14). Moreover, in the foodborne pathogens enterohemorrhagic Escherichia coli O157:H7 (EHEC) and Salmonella enterica serovar Typhimurium, EutR regulates expression of virulence traits (5, 13, 15, 16). Despite the continual replenishment of EA in the GI tract, it has been reported that commensal bacteria do not utilize EA (17), and thus, EA utilization is a trait associated with pathogenesis (3, 4).

**FIG 1:**
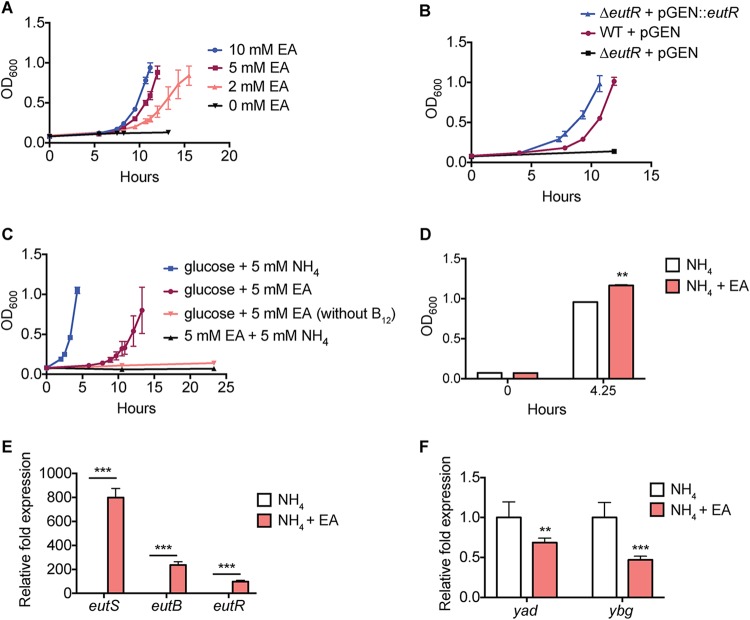
EA-dependent growth and signaling in E. coli HS. (A) Growth curve of HS grown in minimal medium with indicated EA concentrations. *n* = 3. OD_600_, optical density at 600 nm. (B) Growth curve of wild type (WT) with empty vector, Δ*eutR* mutant with empty vector, and *eutR* complemented strain grown in minimal medium containing EA. *n* = 3. (C) Growth curve of HS grown in minimal medium with indicated carbon and nitrogen sources or without vitamin B_12_, as specifically indicated. *n* = 3. (D) Bacterial cell density at indicated time points after growth in minimal medium with NH_4_ or NH_4_ and EA. *n* = 3. (E) Reverse transcription-quantitative PCR (qRT-PCR) of *eut* gene expression in HS grown in in minimal medium with NH_4_ or NH_4_ and EA. *n* = 3. (F) qRT-PCR of fimbrial genes in HS grown in minimal medium with NH_4_ or NH_4_ and EA. *n* = 6. For all, unless indicated, vitamin B_12_ was added whenever the medium was supplemented with EA. Error bars represent the mean ± standard deviation (SD). **, *P *≤* *0.01; ***, *P* < 0.001.

The idea that EA is a noncompetitive metabolite for pathogens is largely perpetuated by data that showed that commensal E. coli isolated from ruminants did not consume EA in a modified bovine intestinal fluid (17). However, subsequent genome sequencing revealed that at least one of the E. coli strains used in the study contained several single nucleotide polymorphisms (SNPs) and an insertion element in the *eut* operon (18), which is expected to render this strain unable to utilize EA. In contrast, the *eut* operon of the human commensal E. coli HS strain contains an intact *eut* locus (19). HS was isolated from the stool of a healthy laboratory scientist and is used as a representative of nondomesticated E. coli in a number of human colonization studies (19–21). Therefore, to revisit EA utilization by human commensal E. coli, we assessed growth of HS when cultured in a minimal medium containing EA as the sole nitrogen or carbon source. Physiologically relevant concentrations of EA supported EutR-dependent growth of HS when provided as a nitrogen (but not carbon) source (Fig. 1A to C). Similarly to other E. coli strains, growth on EA required the addition of vitamin B_12_ (Fig. 1C).

The GI tract contains several nitrogen sources that might diminish the potential importance of EA utilization in HS. To test this, we measured growth of HS in minimal medium containing NH_4_ only or NH_4_ and EA. When EA was added as a supplement to the medium, HS grew to a higher cell density than it did in medium containing only NH_4_ (Fig. 1D). In support of these data, we also measured a significant increase in *eut* gene expression from HS grown in medium supplemented with EA compared to medium without EA supplementation (minimal medium containing NH_4_ or Dulbecco’s modified Eagle’s medium) (Fig. 1E; see also Fig. S1 in the supplemental material). To confirm that EA utilization by a human E. coli isolate was not unique to the HS isolate, we next examined EA utilization in E. coli Nissle, which was isolated from the stool of a German soldier during World War I (22, 23). Consistent with the HS data, Nissle grew and responded to EA (Fig. S2A to D). Altogether, these data indicate that human commensal E. coli strains have maintained the ability to sense and utilize EA as a metabolite and that EA enhances growth in the presence of alternative nitrogen sources (as would be found in the gut).

10.1128/mBio.01429-18.2 FIG S1 qRT-PCR of *eut* gene expression in E. coli HS grown in Dulbecco’s modified Eagle’s medium (DMEM) without or with EA and vitamin B_12_. *n* = 3; error bars represent the mean ± SD. **, *P* < 0.01. Download FIG S1, TIF file, 0.14 MB. Copyright © 2018 Rowley et al. 2018 Rowley et al. This content is distributed under the terms of the Creative Commons Attribution 4.0 International license.

10.1128/mBio.01429-18.3 FIG S2 EA-dependent growth and signaling in E. coli Nissle. (A) Growth curve of Nissle grown in minimal medium with indicated EA concentrations and vitamin B_12_. (B) Bacterial cell density at indicated time points after growth in minimal medium with NH_4_ or with NH_4_, EA, and vitamin B_12_. (C) qRT-PCR of *eut* gene expression in HS grown in DMEM without or with EA and vitamin B_12_. (D) qRT-PCR of *eut* gene expression in Nissle grown in minimal medium containing NH_4_ or EA and vitamin B_12_. For all, *n* equals 3; error bars represent the mean ± SD. *, *P* < 0.05; **, *P *≤* *0.01; ***, *P ≤ *0.001. Download FIG S2, TIF file, 0.31 MB. Copyright © 2018 Rowley et al. 2018 Rowley et al. This content is distributed under the terms of the Creative Commons Attribution 4.0 International license.

We previously reported that EA influences expression of genes carried outside the *eut* locus in EHEC and *Salmonella*, including expression of fimbriae (5, 13, 15, 16). HS and EHEC share a conserved set of fimbrial loci; therefore, we next measured expression of one gene in each of the conserved loci (expression of eight genes was measured) in HS grown in minimal medium with NH_4_ only or NH_4_ and EA. We measured an ∼2- and 3-fold change in expression of genes carried in the *yad* and *ybg* loci, respectively (Fig. 1F). Interestingly, EA supplementation resulted in reduced levels of fimbrial gene expression in HS, which is the opposite of the impact of EA on EHEC fimbrial gene expression. These differences in expression may be reflective of the different colonization niches of these strains (lumen/mucus [HS] versus epithelial attachment [EHEC]). Regardless, these findings provide proof-of-principle data that similarly to EA-dependent growth, EA-dependent signaling is conserved in human commensal E. coli and not restricted to pathogens.

**FIG 2:**
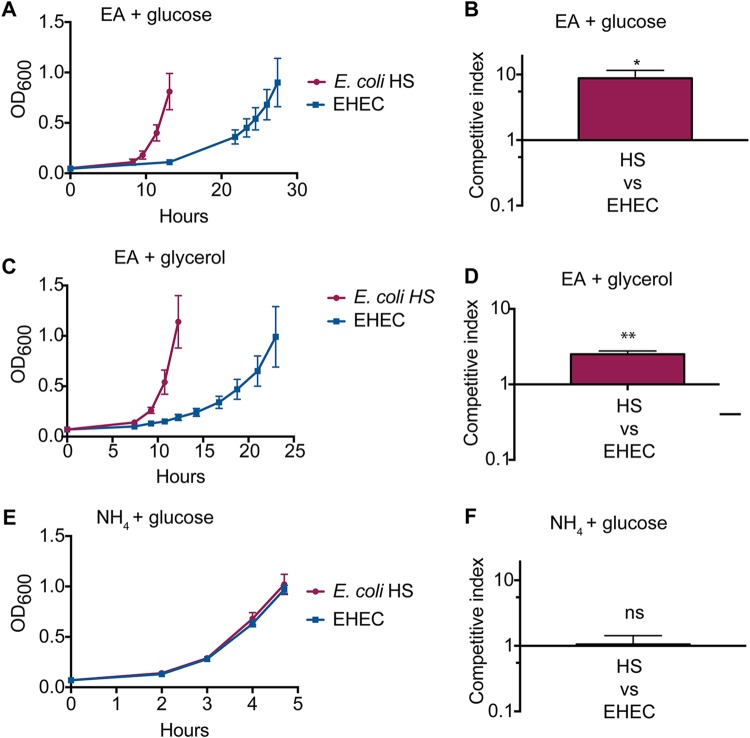
HS outcompetes EHEC specifically during growth on EA. (A) Growth curve of E. coli HS and EHEC in minimal medium with EA and glucose. (B) Competition assay between E. coli HS and EHEC in minimal medium with EA and glucose. (C) Growth curve of E. coli HS and EHEC in minimal medium with EA and glycerol. (D) Competition assay between E. coli HS and EHEC in minimal medium with EA and glycerol. For panels A to D, vitamin B_12_ was added to the medium. (E) Growth curve of E. coli HS and EHEC in minimal medium with NH_4_ and glucose. (F) Competition assay between E. coli HS and EHEC in minimal medium with NH_4_ and glucose. For all, *n* equals 3; error bars represent the mean ± standard deviation. *, *P *≤* *0.05; **, *P *≤* *0.01; ns, *P *>* *0.05.

Scavenging nutrients is paramount for success in colonizing the host intestinal niche (24, 25). Commensal E. coli and EHEC compete for similar resources (24), and EA has been proposed to provide a selective growth advantage to EHEC over commensal E. coli (17). Therefore, we next compared growth of HS and EHEC in EA-minimal medium (containing glucose as the carbon source). Surprisingly, HS grew more rapidly than EHEC when EA was provided as the sole nitrogen source (Fig. 2A), with a doubling time of 1.6 h compared to 4.3 h, respectively (of note, the doubling time of Nissle was 1.3 h [Fig. S2A]). Consistent with these data, during competition HS was recovered at nearly 10-fold-higher levels than EHEC (Fig. 2B). *eut* expression and/or enzymatic activity may be subject to carbon catabolite repression (26, 27); therefore, it is possible that effectiveness of carbon catabolite repression between HS and EHEC caused the differences in growth rates. To test this idea, we repeated the growth and competition experiments in EA-minimal medium containing glycerol as the sole carbon source. During exponential growth, growth rates of HS and EHEC were similar to growth rates in medium containing glucose, with doubling times of 1.4 h and 4.2 h, respectively (Fig. 2C). Of note, we observed a slightly shorter lag phase for EHEC grown in EA-minimal medium containing glycerol compared to glucose. Even so, consistent with the previous assay, HS was recovered in higher numbers than EHEC during competition (>2-fold) (Fig. 2D). Interestingly, this growth advantage was specific for EA utilization as no differences in bacterial growth or recovery were measured when HS and EHEC were cultured in minimal medium containing NH_4_ as the sole nitrogen source (Fig. 2E and F and Fig. S3A and B).

10.1128/mBio.01429-18.4 FIG S3 (A) Growth curve of E. coli HS and EHEC in minimal medium with NH_4_ and glycerol. (B) Competition assay between E. coli HS and EHEC in minimal medium with NH_4_ and glycerol. For all, *n* equals 3; error bars represent the mean ± SD. ns, *P *> 0.05. Download FIG S3, TIF file, 0.11 MB. Copyright © 2018 Rowley et al. 2018 Rowley et al. This content is distributed under the terms of the Creative Commons Attribution 4.0 International license.

Although genes encoding EA utilization are carried by phylogenetically diverse bacteria (27), EA utilization has been suggested to be a potential virulence determinant and/or has been specifically linked to pathogenesis (i.e., references 4, 7, and 28 to 31). Our findings reveal that commensal GI bacteria rely on EA to enhance growth, and thus, EA utilization and signaling are more complex than previously appreciated. This work suggests that further investigation on the impact of EA utilization on host-microbiota-pathogen interaction is warranted.

10.1128/mBio.01429-18.1 TEXT S1 Supplemental materials and methods. Download Text S1, PDF file, 0.05 MB. Copyright © 2018 Rowley et al. 2018 Rowley et al. This content is distributed under the terms of the Creative Commons Attribution 4.0 International license.

10.1128/mBio.01429-18.5 TABLE S1 Oligonucleotides used in this study. Download Table S1, PDF file, 0.02 MB. Copyright © 2018 Rowley et al. 2018 Rowley et al. This content is distributed under the terms of the Creative Commons Attribution 4.0 International license.
