# Supplementary figures and images for: Ethanolamine Influences Human Commensal Escherichia coli Growth, Gene Expression, and Competition with Enterohemorrhagic E. coli O157:H7
Source: mBio. 2018 Oct 2;9(5):e01429-18. doi: 10.1128/mBio.01429-18 (PMC6168858; doi:10.1128/mBio.01429-18)

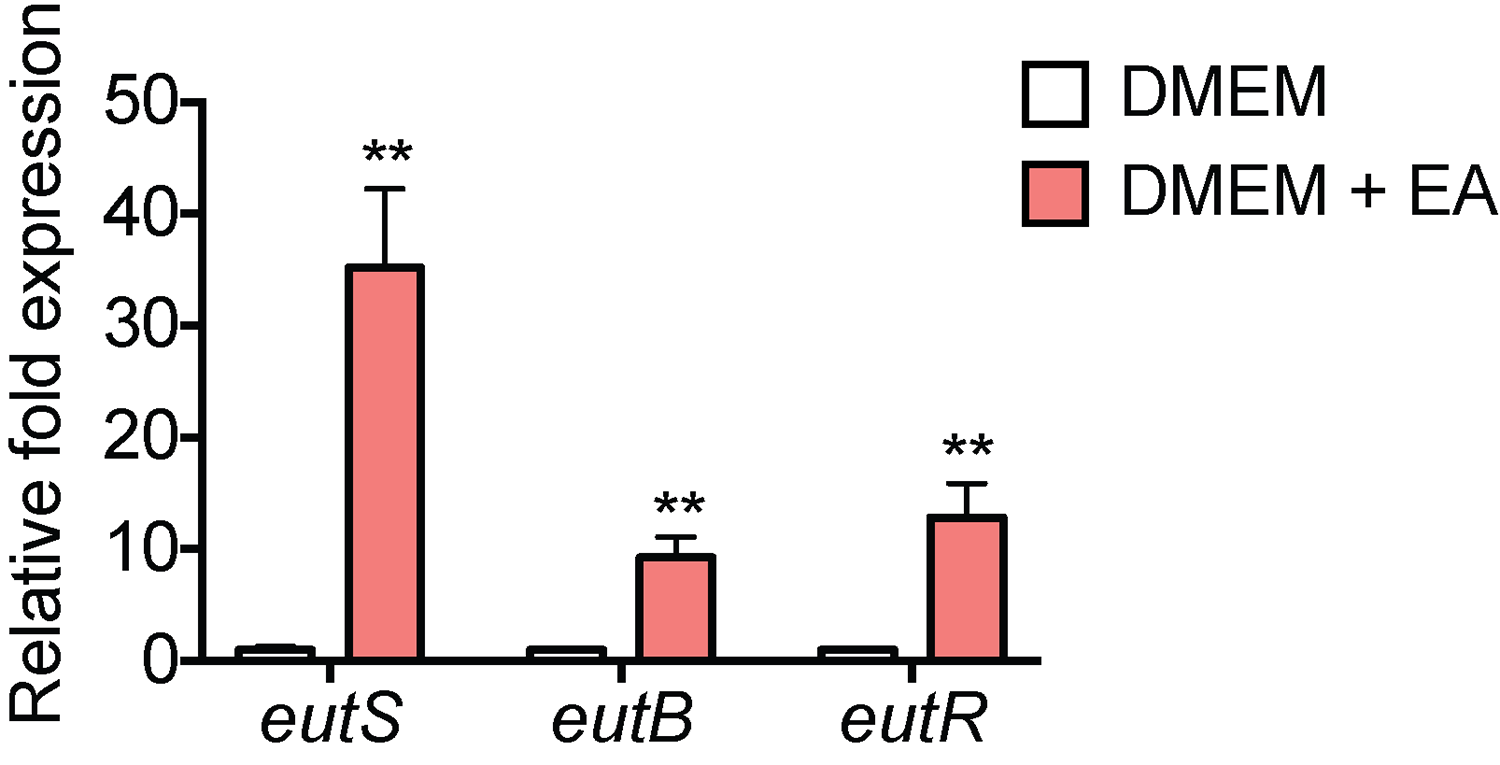

Supplement: FIG S1 [file mbo005184085sf1.tif]

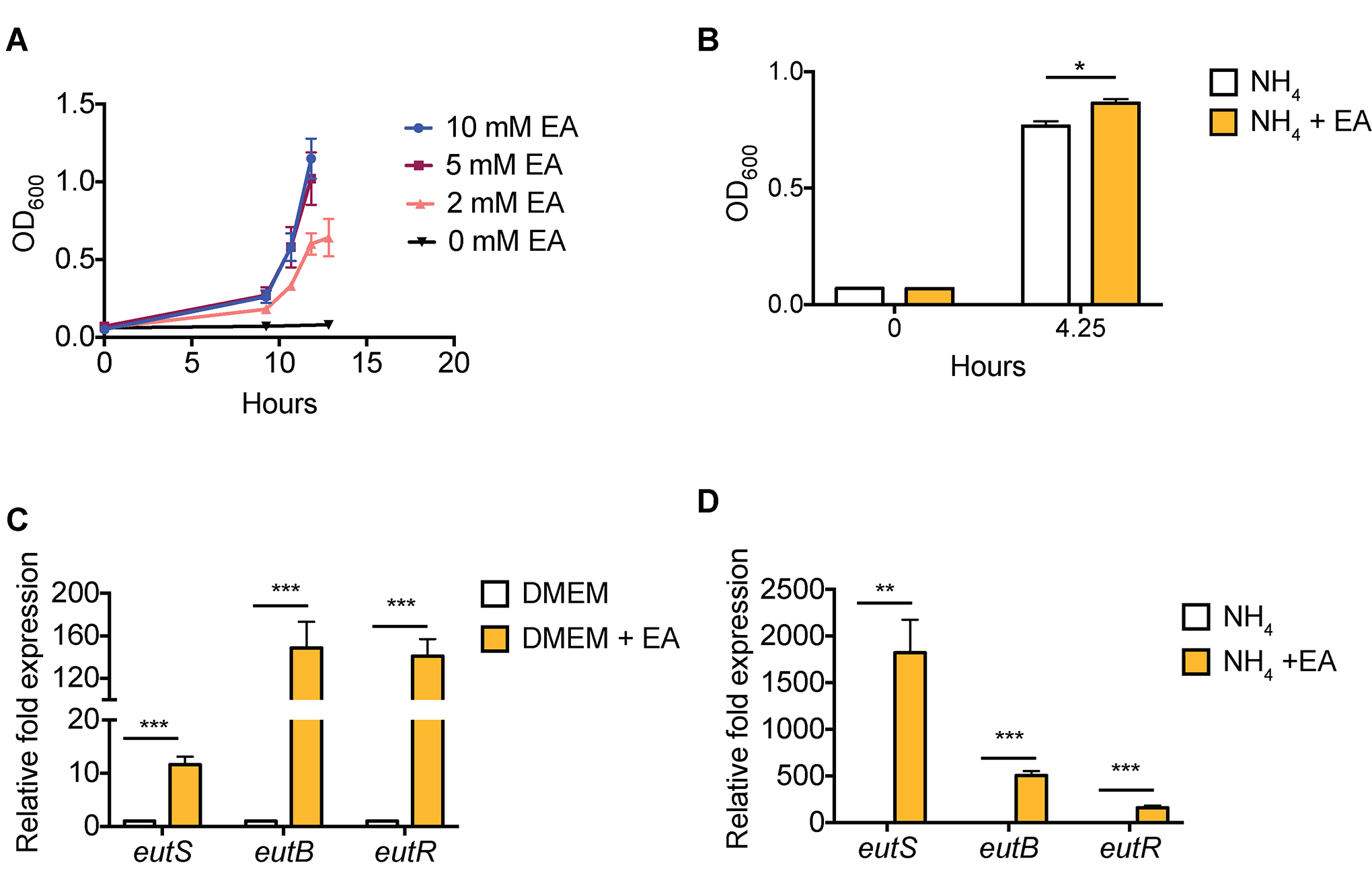

Supplement: FIG S2 [file mbo005184085sf2.tif]

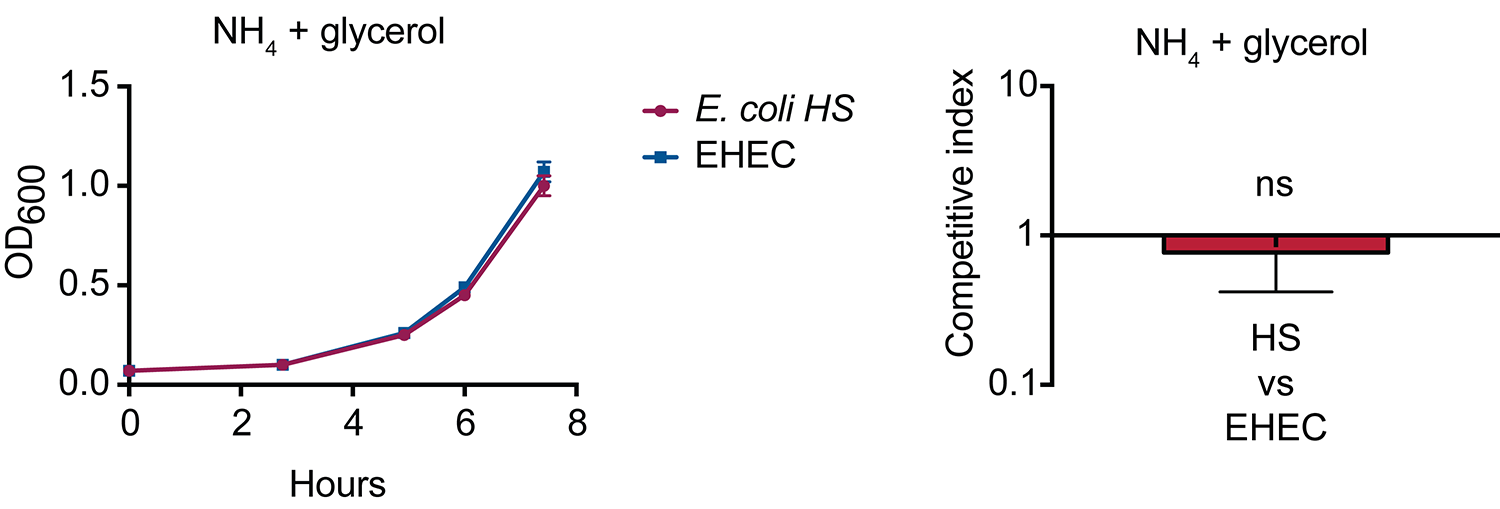

Supplement: FIG S3 [file mbo005184085sf3.tif]
