# Supplementary material for: Ethanolamine Influences Human Commensal Escherichia coli Growth, Gene Expression, and Competition with Enterohemorrhagic E. coli O157:H7
Source: mBio. 2018 Oct 2;9(5):e01429-18. doi: 10.1128/mBio.01429-18 (PMC6168858; doi:10.1128/mBio.01429-18)
Supplement: TEXT S1 [file mbo005184085s1.pdf]

MATERIALS AND METHODS

Strains, plasmids, and recombinant DNA. *E. coli* HS (1), *E. coli* Nissle (2), and *E. coli* 86-24 (EHEC) (3) were used in this study. Bacteria were grown overnight (O/N) shaking in Luria-Bertani (LB) at 37°C. For growth curves, O/N cultures were washed and resuspended in PBS and then diluted 1:100 into the indicated growth medium. M9 minimal medium (4) was prepared without the addition of a nitrogen source to the minimal salts as previously described (5). Briefly, this medium (1 L) was made by adding 200 mL of concentrated M9 salts (containing per liter: 64 g Na_2HPO_4-$7H_2O$; 15 g KH_2PO_4; 2.5 g NaCl); 2 mL 1 M $MgSO_4$, 100 µl 1 M $CaCl_2$, and 5 mg thiamine. Glucose was typically added as the carbon source (0.4% final concentration). When indicated, glycerol was added as the carbon source (0.4% final concentration). The nitrogen sources (Sigma) were ethanolamine (EA) hydrochloride and NH_4Cl. Dulbecco's Modified Eagle Medium was purchased from Invitrogen. Unless indicated, 150 nM vitamin B_{12} was added to the medium whenever EA was added (cyanocobalamin; Sigma).

An *eutR* deletion strain of *E. coli* HS (strain designation: MK85) was generated using λ-red mutagenesis (6). Briefly, PCR products (using primers listed in Table S1) were amplified from plasmid pKD4 with flanking regions matching *eutR* and transformed into *E. coli* HS expressing the Red genes from plasmid pKD46. The resistance cassette was resolved with flippase from temperature-sensitive plasmid pCP20, which was subsequently cured by growing at 42°C. The deletion was confirmed by sequencing. The *eutR* mutant was complemented with plasmid pCAR001. pCAR001 was

constructed by amplifying *E. coli* HS genomic DNA using primers specific to the *eutR* gene, including 206 nucleotides upstream of the ATG start site (using primers listed in Table S1). Amplified DNA was digested with NheI and SacI and inserted into pGEN-MCS (7) (Addgene MTA). As a control, the WT and Δ*eutR* strains were transformed with the empty pGEN-MCS vector.

Growth and competition experiments. Bacteria were grown aerobically in minimal medium with 5 mM EA or 5 mM NH_4Cl, unless otherwise indicated. Doubling time was calculated as ln(2) / growth rate during linear growth phase. For competition experiments, equal numbers of bacterial colony forming units (cfu) were added to minimal medium supplemented with EA or NH_4Cl. For glucose cultures, samples were collected at 25 h (EA) or 3.5 h (NH_4Cl), time points that were reflective of EHEC at mid-log growth phase as determined by pure culture growth curves. For glycerol cultures, samples were collected at 8.75 (EA) or 6 h (NH_4Cl). Samples were serially diluted and plated on LB with no selection to enumerate total cfu or LB with streptomycin to enumerate EHEC cfu. *E. coli* HS cfu were determined by subtracting EHEC cfu from total cfu. The competitive index was calculated by dividing *E. coli* HS by EHEC cfu.

RNA extraction and quantitative reverse transcription polymerase chain reaction (qRT-PCR)

Bacteria were grown in DMEM for 6 h statically under a 5% CO_2 atmosphere without or with EA supplementation (5 mM) or aerobically in minimal medium containing 2 mM NH_4Cl or 2 mM NH_4Cl and 5 mM EA to an $O.D._{600}$ of 0.3-0.4. Then bacterial cells were

suspended in Trizol (Life Technologies), and RNA was extracted using the RiboPure Bacteria RNA isolation kit (Ambion). Primers used in real-time qRT-PCR assays were validated prior to use and are listed in Table S1. Reaction mixtures were prepared as previously described (8). qRT-PCR was performed using a one-step reaction in an ABI 7500-FAST sequence detection system (Applied Biosciences). All data were normalized to the levels of *rpoA* and analyzed using the comparative cycle threshold (C_T) method (9).

Statistical analyses. For growth curves and gene expression experiments, statistical significance was determined using Student's *t* test, and a P value of ≤ 0.05 was considered significant. For the competition experiments, statistical significance was determined by one-sample *t* test with an expected value of 1. All experiments were repeated at least twice and with three biological replicates.

SUPPLEMENTAL REFERENCES

1. Levine MM, Bergquist EJ, Nalin DR, Waterman DH, Hornick RB, Young CR, Sotman S, Rowe B. 1978. *Escherichia coli* strains that cause diarrhoea but do not produce heat-labile or teat-stable enterotoxins and are non-invasive. Lancet i:1119-1122.
2. Sonnenborn U. 2016. *Escherichia coli* strain Nissle 1917-from bench to bedside and back: history of a special Escherichia coli strain with probiotic properties. FEMS Microbiol Lett 363:pii: fnw212.

3. Griffin PM, Ostroff SM, Tauxe RV, Greene KD, Wells JG, Lewis JH, Blake PA. 1988. Illnesses associated with *Escherichia coli* O157:H7. Ann Intern Med 109:705-712.

4. Sambrook J, Fritsch EF, Maniatis T. 1989. Molecular cloning: a laboratory manual, 2 ed. Cold Spring Harbor Laboratory Press, Cold Spring Harbor, NY.

5. Kendall MM, Gruber CC, Parker CT, Sperandio V. 2012. Ethanolamine controls expression of genes encoding components involved in interkingdom signaling and virulence in enterohemorrhagic *Escherichia coli* O157:H7. mBio 3:e00050-12.

6. Datsenko KA, Wanner BL. 2000. One-step inactivation of chromosomal genes in *Escherichia coli* K-12 using PCR products. Proc Natl Acad Sci 97:6640-6645.

7. Lane MC, Alteri CJ, Smith SN, Mobley HLT. 2007. Expression of flagella is coincident with uropathogenic *Escherichia coli* ascension to the upper urinary tract. Proc Natl Acad Sci 104:16669-16674.

8. Kendall MM, Rasko D, A., Sperandio V. 2010. The LysR-type regulator QseA regulates both characterized and putative virulence genes in enterohaemorrhagic *Escherichia coli* O157:H7. Mol Microbiol 76:1306-1321.

9. Livak KJ. 1997. ABI Prism 7700 Sequence Detection System. User Bulletin no 2, PE Applied Biosystems 4303859B:777802-778002.
