# Supplementary material for: Ethanolamine Influences Human Commensal Escherichia coli Growth, Gene Expression, and Competition with Enterohemorrhagic E. coli O157:H7
Source: mBio. 2018 Oct 2;9(5):e01429-18. doi: 10.1128/mBio.01429-18 (PMC6168858; doi:10.1128/mBio.01429-18)
Supplement: TABLE S1 [file mbo005184085st1.pdf]

Table S1. Oligonucleotides used in this study.

Primer name	Sequence	Primer use
HS_LR_eutR_F1	CCCGGCATTAACATCATGAAAAAGACC CGTACAGCCAATTTGCACCATCTTTGTG TAGGCTGGAGCTGCTTC	λ-Red
HS_LR_eutR_R1	AAGTGAGTTTATTAAGGTCAGGGATTGG GTGTAACTCCCTCACCCCCACTCTCATA TGAATATCCTCCTTAG	λ-Red
pGEN_HS_eutR_F	CTAGGAGCTCTGTTGGCGCTGTTAACA TCA	*E. coli* HS *eutR* complement
pGEN_HS_eutR_R	CTAGGCTAGCTCACCCCCACTCCCGCA TCC	*E. coli* HS *eutR* complement
HS_rpoA_F	CGCGGTCGTGGTTATGTG	qRT-PCR
HS_rpoA_R	GCGCTCATCTTCTTCCGAAT	qRT-PCR
HS_eutS_F	GGCGGCGACTCATGGATAAA	qRT-PCR
HS_eutS_R	TTCTTCGCCAGTTCCTCACC	qRT-PCR
HS_eutB_F	GCGTGGCGGCAAAGC	qRT-PCR
HS_eutB_R	CACCGGATTATTGCGGATGT	qRT-PCR
HS_eutR_F	ATCCGGGCAAGTTTCATGGT	qRT-PCR
HS_eutR_R	CGGAATGCCAAACCAGAACG	qRT-PCR
Nissle_rpoA_F	CGCGGTCGTGGTTATGTG	qRT-PCR
Nissle_rpoA_R	GCGCTCATCTTCTTCCGAAT	qRT-PCR
Nissle_eutS_F	CCGGATGCGGGCGCAATCGG	qRT-PCR
Nissle_eutS_R	CGATATGCACATCGGCGGCT	qRT-PCR
Nissle_eutB_F	GCGTGGCGGCAAAGC	qRT-PCR
Nissle_eutB_R	CACCGGATTATTGCGGATGT	qRT-PCR
Nissle_eutR_F	ATCCGGGCAAGTTTCATGGT	qRT-PCR
Nissle_eutR_R	CGGAATGCCAAACCAGAACG	qRT-PCR
HS_yad_RT_F1	AGGTTTTGCCCTGTCTGGTC	qRT-PCR
HS_yad_RT_R1	TGCAGCAGGATCAGACCATC	qRT-PCR
HS_ybg_RT_F1	AGATATCGACCTGGCTCCGA	qRT-PCR
HS_ybg_RT_R1	GGTTGCATTCGCCGTAACTG	qRT-PCR
